# Identification of tissue sections from decellularized liver scaffolds for repopulation experiments^☆^

**DOI:** 10.1016/j.heliyon.2021.e06129

**Published:** 2021-02-13

**Authors:** Philipp Felgendreff, Claudia Schindler, Franziska Mussbach, Chichi Xie, Felix Gremse, Utz Settmacher, Uta Dahmen

**Affiliations:** a Department of General, Visceral and Vascular Surgery, University Hospital Jena, Jena, Germany; b Research Program “Else Kröner-Forschungskolleg AntiAge”, Jena University Hospital, Jena, Germany; c Institute for Experimental Molecular Imaging, RWTH Aachen University, Aachen, Germany

**Keywords:** Decellularization, Liver engineering, Organ engineering, Extracellular matrix

## Abstract

**Background:**

Biological organ engineering is a novel experimental approach to generate functional liver grafts by decellularization and repopulation. Currently, healthy organs of small or large animals and human organs with preexisting liver diseases are used to optimize decellularization and repopulation.

However, the effects of morphological changes on allo- and xenogeneic cell-scaffold interactions during repopulation procedure, e.g., using scaffold-sections, are unknown. We present a sequential morphological workflow to identify murine liver scaffold-sections with well-preserved microarchitecture.

**Methods:**

Native livers (CONT, n = 9) and livers with experimentally induced pathologies (hepatics steatosis: STEA, n = 7; hepatic fibrosis induced by bile duct ligation: BDL, n = 9; nodular regenerative hyperplasia induced by 90% partial hepatectomy: PH, n = 8) were decellularized using SDS and Triton X-100 to generate cell-free scaffolds. Scaffold-sections were assessed using a sequential morphological workflow consisting of macroscopic, microscopic and morphological evaluation: (1) The scaffold was evaluated by a macroscopic decellularization score. (2) Regions without visible tissue remnants were localized for sampling and histological processing. Subsequent microscopical examination served to identify tissue samples without cell remnants. (3) Only cell-free tissue sections were subjected to detailed liver-specific morphological assessment using a histological and immunohistochemical decellularization score.

**Results:**

Decellularization was feasible in 33 livers, which were subjected to the sequential morphological workflow. In 11 of 33 scaffolds we achieved a good macroscopic decellularization result (CONT: 3 scaffolds; STEA: 3 scaffolds; BDL: 3 scaffolds; PH: 2 scaffolds). The microscopic assessment resulted in the selection of 88 cell-free tissue sections (CONT: 15 sections; STEA: 38 sections; BDL: 30 sections; PH: 5 sections). In 27 of those sections we obtained a good histological decellularization result (CONT: 3 sections; STEA: 6 sections; BDL: 17 sections; PH: 1 section). All experimental groups contained sections with a good immunohistochemical decellularization result (CONT: 6 sections; STEA: 5 sections; BDL: 4 sections; PH: 1 section).

**Discussion:**

Decellularization was possible in all experimental groups, irrespectively of the underlying morphological alteration. Furthermore, our proposed sequential morphological workflow was suitable to detect tissue sections with well-preserved hepatic microarchitecture, as needed for further repopulation experiments.

## 1 Introduction

The major problem in liver transplantation surgery is the increasing shortage of donor organs. In 2019, more than 1330 patients with end-stage liver disease were waiting on the EUROTRANSPLANT list for transplantation. In contrast, only 850 liver transplantations were performed due to the lack of donor organs with good quality.

Biological organ engineering is a promising experimental approach to solve the clinical problem of donor shortage by generating functional liver grafts. The liver organ engineering concept is based on the process of decellularization and repopulation of organs taking advantage of their preserved natural structure [1]. During decellularization, all cellular material must be removed from the donor liver to obtain a cell-free organ scaffold without losing the integrity of the liver-specific microarchitecture. This decellularization process is performed by perfusing the liver with ionic or non-ionic detergents, resulting in a cell-free scaffold. During the repopulation process, the cell-free scaffold must be reseeded with either organ-specific parenchymal and non-parenchymal cells, progenitor cells or stem cells to generate a potentially functional organ [2, 3].

Currently, organs from small and large animals [4], as well as human organs [5], are used to optimize the decellularization and repopulation procedure.

Regarding the decellularization procedure, special attention was given to the choice and application of detergent. Different detergents (such as sodium dodecyl sulfate (SDS) [6] or ammonium hydroxide [7]), different perfusion modes (such as constant or oscillation perfusion pressure [8]) and different application routes (such as the portal vein or hepatic artery [9]) were evaluated.

Furthermore, the mode and route of cell application are central factors influencing repopulation of vascular and sinusoidal structures of the liver scaffold.

The first promising results were obtained by using single-step endothelial cell and multi-step parenchymal cell application via the portal vein and hepatic vein or hepatic artery [10]. So far, long-term parenchymal and endothelial in-vitro function was neither established in the xenogeneic nor in the allogeneic model. There is still a number of open questions to be addressed.

The ideal source for the organ scaffold as well as for the repopulating cells still needs to be identified. Until now, only young, healthy organs without any signs of disease were used for the xenogeneic animal models.

However, when considering the use of allogeneic human organs, it becomes evident that healthy organs are not and will not be available for organ engineering.

Up to now, the impact of the allo- or xenogeneic nature of the scaffold on repopulation, function, and long-term acceptance is not fully elucidated. However, the extracellular matrix from different species varies in terms of the molecular composition (e.g., glycosaminoglycan content, hydroxyproline and proline content) and matrix proteins (e.g., fibronectin, cell adhesion molecules (CAM) and proteoglycans).

Therefore, it is highly likely that cell-scaffold interactions are influenced by species differences as well as by the type of cell used for reseeding and the preservation of the matrix proteins [11].

Only few studies focused on the interaction of cell and native matrix. Hence, the influence of cell-matrix interaction on cell adherence, viability, and function, considering the species as well as the cell type, is widely unknown.

For a better understanding of cell-scaffold interaction, it is crucial to study the impact of species differences and structural impairment – especially due to minor hepatic diseases – on cell adhesion, proliferation and function.

In order to assess the influence of these factors in an easy to handle cell culture model, we and others propose the use of cell-free scaffold-sections [12] enabling high comparability of repopulation experiments.

The currently available molecular and morphological analysis of the decellularization result is based on qualitative as well as quantitative parameters.

The quantitative parameters of the molecular analysis are clearly defined and include the determination of the remnant deoxyribonucleic acid content [6, 13, 14], but also the quantification of matrix components such as glycosaminoglycan [13, 15, 16, 17] and matrix proteins such as elastin [18]; collagen [13, 15, 16]; using homogenized scaffold samples.

In contrast, qualitative parameters for morphological analysis are much less defined, resulting in a rather global assessment of the liver-specific microarchitecture to determine the decellularization result [19].

Both analytical approaches are not suitable to determine morphological differences in the scaffold microarchitecture.

The aim of this study was to establish a sequential morphological workflow to assess scaffold microarchitecture of decellularized murine scaffolds with minor histological impairment (hepatic steatosis, liver fibrosis and nodular regenerative hyperplasia) as model system. This workflow should serve to evaluate the quality of the decellularized scaffold in order to identify cell-free sections with preserved liver-specific microarchitecture for further repopulation experiments.

## 2 Materials and methods

### 2.1 Study design and sample size

**Figure 1:**
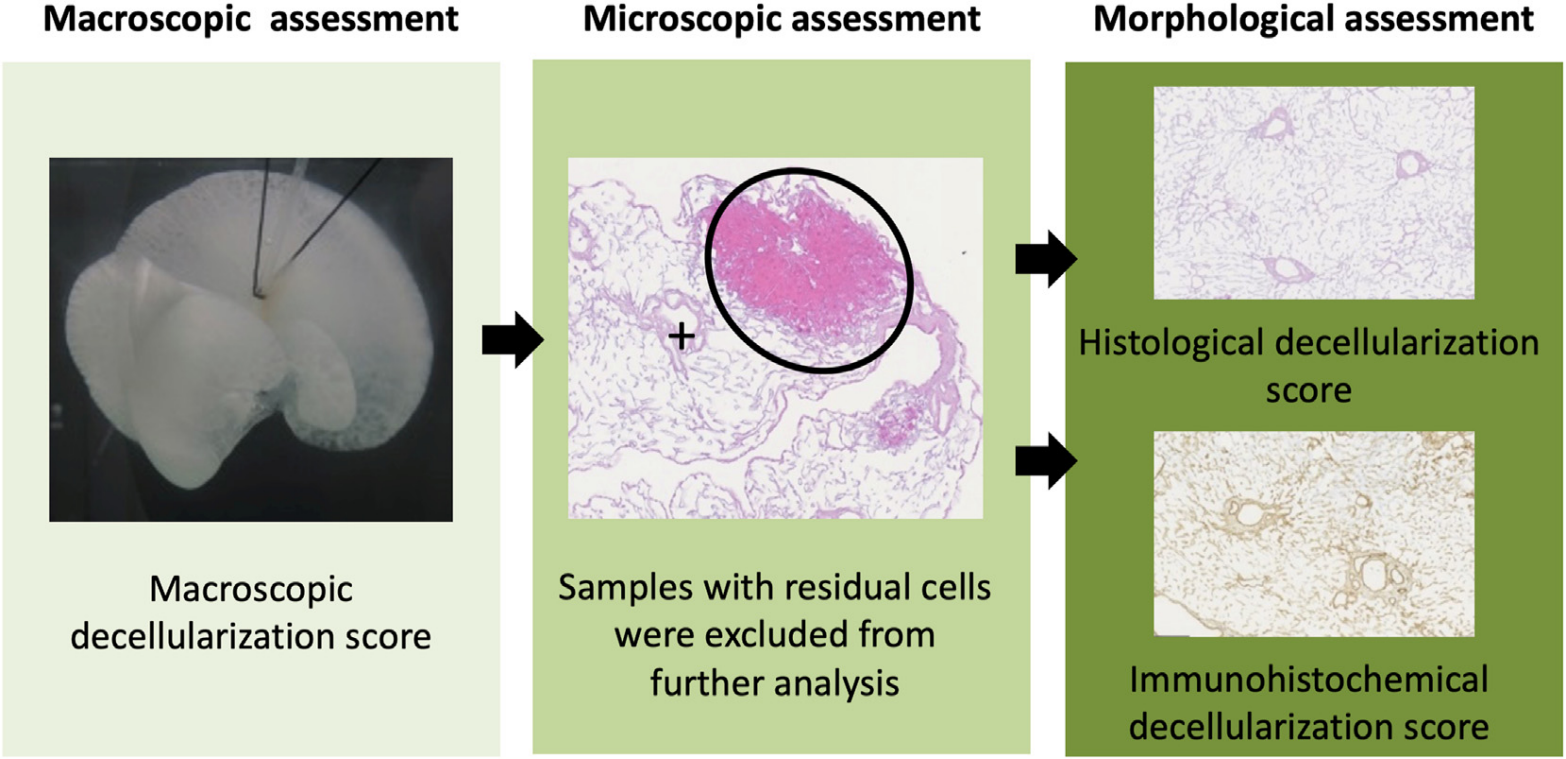
Sequential morphological workflow: The sequential morphological workflow was used for all decellularized organs to identify sections with preserved liver specific microarchitecture. In the first step, all decellularized organs were macroscopic assessed using a macroscopic decellularization score to judge the overall scaffold quality and to localize regions without tissue remnants for further sampling. In the second step, scaffold samples without macroscopically visible tissue remnants were microscopic assessed to identify scaffold-sections without cell remnants. In the third step, cell-free tissue sections were subjected to a morphological assessment of hepatic microarchitecture consisting of a histological and immunohistochemical decellularization score.

In order to develop a sequential morphological workflow to identify scaffold-sections for further repopulation experiments, we designed a three-step approach (shown in Figure 1):

1. Macroscopic assessment of the decellularized organ using a macroscopic decellularization score to judge the overall scaffold quality and to localize regions without tissue remnants for further sampling
2. Microscopic assessment of the scaffold-sections obtained from areas without macroscopically visible tissue remnants using hematoxylin and eosin (HE) and Periodic acid-Schiff reaction (PAS)-staining to identify tissue samples without histologically detectable cell remnants
3. Morphological assessment of the scaffold-sections using a
  a. histological decellularization score to grade the preservation level of microarchitecture in the cell-free scaffold-sections after the decellularization procedure
  b. immunohistochemical decellularization score to grade the preservation level of 4 selected matrix proteins in one randomly cell-free selected scaffold-sections per organ after the decellularization procedure

Considering the limited availability of human liver grafts, our exploratory study was performed using 36 wild-type mice (9 mice per group) with experimentally induced liver pathologies. The sample size was calculated using the G∗Power statistics software, based on the study by Caralt *et al.* [20]. For a single factor ANOVA with four experimental groups, an effect size *f* of 0.6 (or Cohens d = 1.2), an α error of 0.05 and a power (1 – β error) of 0.80, the sample size per experimental group is n = 9.

The following three different liver pathologies were experimentally induced and compared to the control group (CONT):

• hepatic steatosis (STEA), by feeding the mice a methionine-choline-deficient and fat-enriched diet (Ssniff, Sulzfeld, Germany) for 4 weeks;
• liver fibrosis and biliary obstruction, via bile duct ligation (BDL) about 4 weeks prior to explantation;
• and nodular regenerative hyperplasia, by 90% partial hepatectomy (PH) about 4 weeks prior to harvesting the liver.

After organ explantation, decellularization was achieved by perfusion with 1% SDS and Triton X-100. All organs were evaluated using the proposed sequential morphological workflow.

In order to demonstrate the integrity of the vascular structure independently of the experimental intervention, the portal venous system was visualized in two additional livers of each group using the computer tomography scanner (CT).

### 2.2 Animals

Male C57BL/6N inbred mice with a body weight of 20–32 g were purchased from Charles River Laboratories (Sulzfeld, Germany). Mice were housed in a certified rodent facility under constant environmental conditions (30–70% air humidity, 19–22 °C temperature, 12-h light/dark cycle). They were fed a normal laboratory chow diet (Altromin GmbH & Co. KG, Lippe, Germany), except the steatotic animals receiving the diet described below, and had free access to water.

### 2.3 Intervention

#### 2.3.1 Steatosis induction – fat diet, (STEA)

The experimental induction of fatty liver was performed by feeding the animals with a methionine-choline-deficient and fat-enriched diet (Ssniff, Sulzfeld, Germany). The diet was administered for 4 weeks to induce moderate liver steatosis prior to harvesting the organ.

### 2.4 Surgical procedures

All surgical interventions were performed under inhalation anesthesia (2 vol. % isoflurane mixed with 0.3 L/min oxygen using an isoflurane vaporizer, Sigma Delta, UK) after analgesic pretreatment with buprenorphine (Temgesic®; 0.05 mg/kg s.c.) applied 0.5 h prior to the skin incision. Briefly, after the induction of anesthesia, systemic heparinization (300 IU/kg body weight Heparin-sodium-25000-ratiopharm®, Ratiopharm, Germany) was performed. After completion of the respective operative procedure, animals were placed on a heating pad during the recovery period. For postoperative analgesic treatment, buprenorphine was applied subcutaneously twice daily for 3 postoperative days at a dose of 0.05 mg/kg body weight.

#### 2.4.1 Fibrosis induction – bile duct ligation, (BDL)

A laparotomy was performed via a transverse abdominal incision. After the exploration of the liver, the bile duct was identified and mobilized. The bile duct was ligated twice and transected between these two ligatures. After checking for biliary leakage, the abdomen was closed with a two-layer running suture (6-0 Prolene, Ethicon, USA). Mice were observed for at least 4 weeks prior to explanting the liver.

#### 2.4.2 Nodular regenerative hyperplasia induction – extended 90% partial hepatectomy, (PH)

The abdominal cavity was fully exposed as described above. For the mobilization of the liver, the triangular ligament was dissected, and the bowel was exenterated and placed to the left of the animal. Following the resection of the left lateral lobe, the median lobe was removed using the piercing technique described by Madrahimov *et al.* [21]. The removal of the median lobe was performed in two steps by defining a virtual line between the left side of the vena cava and the gallbladder. A clamp was placed 2–3 mm lateral to this line. After removing the left lateral and median lobe, two piercing sutures (6-0 Prolene, Ethicon, USA) were used to close the vessels in the stump. As a next step, the pedicles of the right inferior, superior caudate, and inferior caudate lobe were ligated using silk 6-0 (Ethicon, USA). Finally, the abdomen was irrigated with warm saline solution and closed with a two-layer running suture. After this procedure, mice were observed for 4 weeks postoperatively.

### 2.5 Liver explantation

After placing the anesthetized animals in the supine position, the abdomen was opened via a wide transversal incision. The liver was mobilized by dissecting the triangular ligament and the portal vein was exposed. Two ligatures were placed 1 cm below the liver hilum on the portal vein. The portal vein was cannulated with a 26 gauge catheter (Terumo Europe®, Belgium) that was fixed with two suture lines. Total heparinization was achieved by slowly injecting 300 IU heparin/kg body weight; (Heparin-sodium-25000-ratiopharm®, Ratiopharm, 25000 I.E.) via the portal vein over a period of 10 minutes using a non-peristaltic pump (0.3 ml/min; Perfusor VI, B. Braun, step 7). The diaphragm was opened to place an incision on the suprahepatic vena cava, which allowed the drainage of the perfusate.

After total perfusion of the liver, the organ was freed from the remaining ligaments and removed from the abdominal cavity. This was directly followed by taking a native liver tissue sample, preferably from the inferior caudate liver lobe.

### 2.6 Decellularization

After explanting the liver, the organ was placed in 1% phosphate buffered saline solution and perfused through the portal vein catheter with 1% Triton X-100 for 60 min followed by 1% SDS. A pulsatile flow profile was applied, switching between 1 ml/min and 1.5 ml/min every 4 s, using the Harvard peristaltic pump p70 (Hugo-Sachs, Harvard Apparatus GmbH, Germany). The criteria for termination of the decellularization process were defined based on our experience from previous projects regarding the macroscopic appearance of the acellular liver [12]. We defined to stop the liver perfusion either when no remnant tissue or no macroscopic changes were seen for a period of more than 30 min.

### 2.7 Visualization of the vascular tree

To demonstrate the integrity of the vascular tree irrespectively of experimentally induced pathologies or decellularization procedure, two additional native livers and two scaffolds from each group were injected with radiopaque silicone rubber compound (MICROFIL®, Flow Tech Inc., USA) via the portal vein catheter. The injection procedure was monitored macro- and microscopically throughout the procedure to ensure continuous steady inflow into the liver. Upon curation of the rubber component the contrasted organs were placed in 5% formaldehyde for fixation until CT-scanning using the Tomoscope Duo CT (CT Imaging GmbH, Erlangen, Germany). The CT-scans were performed using the scan protocol HQD-6565-390-90 with 720 projections (approximately 1032 × 1012 pixels) during one full rotation with a scanning time of 90 s. The scans resulted in voxel image representations of the specimens at an isotropic resolution of 70 μm. Further histological workup of these samples was not performed, since the intra-vascular application of the polymerizing agents may cause artefacts disturbing the assessment of the histological sections.

### 2.8 Histological staining

The histological samples were fixed in 5% buffered formalin for 48h and cut (4μm thick) after paraffin embedding. The sections were subjected to routine staining (HE, PAS, Elastica van Gieson (EvG)) following a standard protocol. Sections were digitalized using a Hamamatsu slide scanner (NanoZoomer 2.0 HT, Hamamatsu Electronic Press Co., Ltd., Iwata, Japan).

### 2.9 Immunohistochemical staining

Immunohistochemical staining was performed using a standard protocol. Endogenous peroxidases were blocked using hydrogen peroxide.

Nonspecific binding was prevented by applying Dako Dual Endogenous Enzyme Block (Dako/Agilent; S2003). The following primary polyclonal rabbit antibodies were used: anti-mouse laminin (Dako/Agilent – Z009701-2; 1:1.000 dilution), anti-mouse elastin (Abcam – ab21610; 1:100 dilution), anti-mouse type I collagen (Abcam – ab34710; 1:500 dilution), and anti-mouse type IV collagen (Abcam – ab6586; 1:500 dilution).

The target antigens were visualized using a secondary anti-rabbit antibody labeled with a horseradish peroxidase polymer and DAB as the substrate (Dako Envision + System, HRP, labeled Polymer Anti-Rabbit). Hematoxylin was applied for 7 min as a counterstain for the detection of cell nuclei. Sections were digitalized using a Hamamatsu slide scanner.

### 2.10 Comparison by using the sequential morphological workflow

In the first step of the sequential morphological workflow, all decellularized organs were assessed using the macroscopic decellularization score to judge the overall scaffold quality. Scaffold samples were taken from regions without tissue remnants and subjected to the microscopic evaluation.

In the second step, all samples were assessed microscopically to identify the cell-free scaffold-sections for the further morphological evaluation. For detection of residual cells, all scaffold-sections were stained with HE as well as PAS to identify cell nuclei and residual glycogen components of the cytoplasm.

In the third step, the morphological assessment of the cell-free scaffold-sections was done using a histological and an immunohistochemical decellularization score.

For the histological decellularization score, additional staining of the scaffold-sections with EvG was necessary. The histological decellularization score was used to grade the level of preservation of the microarchitecture based on the presence of vascular structures and the evaluation of vascular integrity. The presence of vascular structures, such as the portal vein, central vein and sinusoidal network, were assessed in the HE-stained sections. The vascular integrity was evaluated in EvG-stained sections by detecting continuous elastic fibers in vascular walls.

The immunohistochemical decellularization score was used to grade the preservation level of 4 selected matrix proteins (collagen I and IV; elastin, laminin) in randomly selected cell-free scaffold-section of each organ. The matrix proteins of the vascular structure were evaluated using collagen I and collagen IV staining. The presence of elastic fibers as well as the basal membrane was investigated using the elastin and laminin staining.

### 2.11 Macroscopic assessment

#### 2.11.1 Macroscopic decellularization score

**Figure 2:**
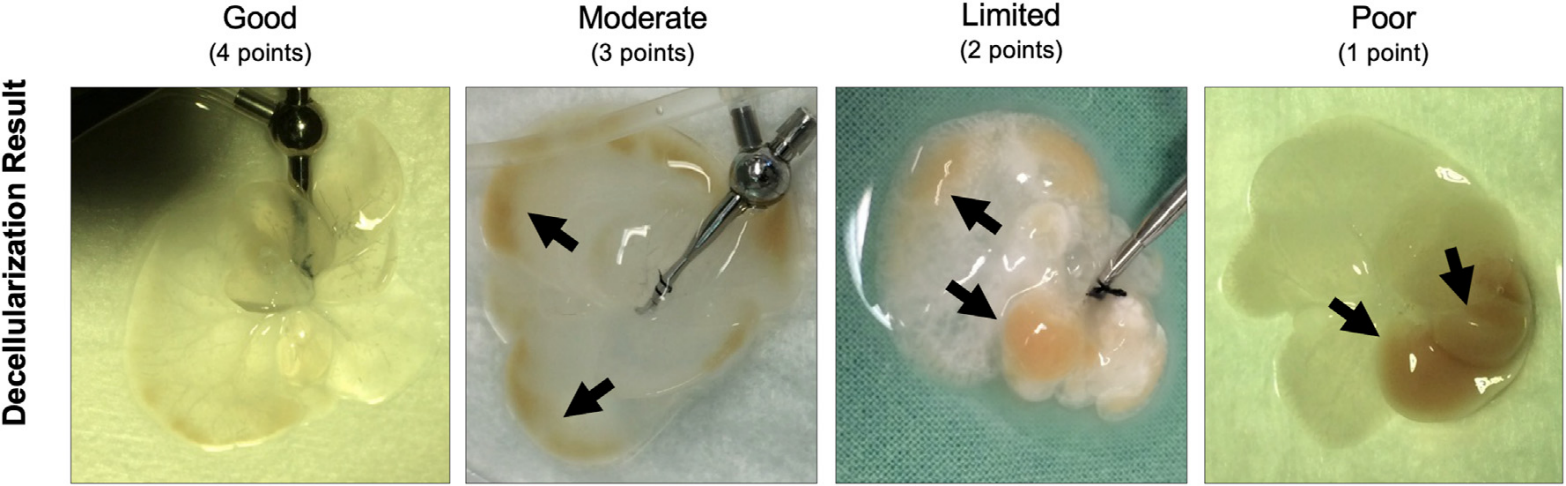
Macroscopic decellularization score: Macroscopic decellularization score was applied on all decellularized organs to identify liver lobes without residual hepatic tissue. The endpoint of decellularization was achieved either when the liver became transparent or no macroscopic changes were seen for a period of more than 30 min. Decellularization judgment: Good decellularization result (4 points), defined by complete removal of hepatic tissue of the whole liver lobe, resulting in fully translucent areas of all liver lobes and allowing the visualization of the whole vascular structure; Moderate decellularization result (3 points), defined by incomplete removal of hepatic tissue in less than half of the parenchyma of a single liver lobe, predominantly at the edges of the liver lobes, resulting in mainly translucent areas of more than half of the respective liver lobe and allowing the visualization of most of the vascular structure; Limited decellularization result (2 points), defined by incomplete removal of hepatic tissue in more than half of a single liver lobe, resulting in individual translucent areas of less than half of the respective liver lobe and allowing the visualization of parts of the vascular structure; Poor decellularization result (1 point), defined by no removal of hepatic tissue within one or more liver lobes, resulting in non-translucent areas of the respective liver lobe and not allowing the visualization of the vascular structure.

The macroscopic decellularization score was used to evaluate all decellularized organs (shown in Figure 2). The score is based on two interrelated criteria: the distribution and extent of remnant hepatic parenchymal tissue and the distribution and extent of translucent areas.

Good decellularization result (4 points), defined by complete removal of hepatic tissue of the whole liver, resulting in fully translucent areas of all liver lobes and allowing the visualization of the whole vascular structure.

Moderate decellularization result (3 points), defined by incomplete removal of hepatic tissue in less than half of the parenchyma of a single liver lobe, predominantly at the edges of the liver lobes, resulting in mainly translucent areas of more than half of the respective liver lobe and allowing the visualization of most of the vascular structure.

Limited decellularization result (2 points), defined by incomplete removal of hepatic tissue in more than half of a single liver lobe, resulting in individual translucent areas of less than half of the respective liver lobe and allowing the visualization of parts of the vascular structure.

Poor decellularization result (1 point), defined by no removal of hepatic tissue within one or more liver lobes, resulting in non-translucent areas of the respective liver lobe and not allowing the visualization of the vascular structure.

### 2.12 Microscopic assessment

The microscopic assessment was applied to all samples without macroscopically visible tissue remnants. To identify scaffold-sections with residual cells, all scaffold-sections were stained with HE as well as PAS. Residual cells were detected using two predefined criteria (presence of residual cell nuclei in HE-stained sections; presence of residual cytoplasmic glycogen in PAS-stained sections). Only sections without evidence of residual cell nuclei or cytoplasmic glycogen were subjected to further analysis.

### 2.13 Morphological assessment

#### 2.13.1 Histological decellularization score

**Figure 3:**
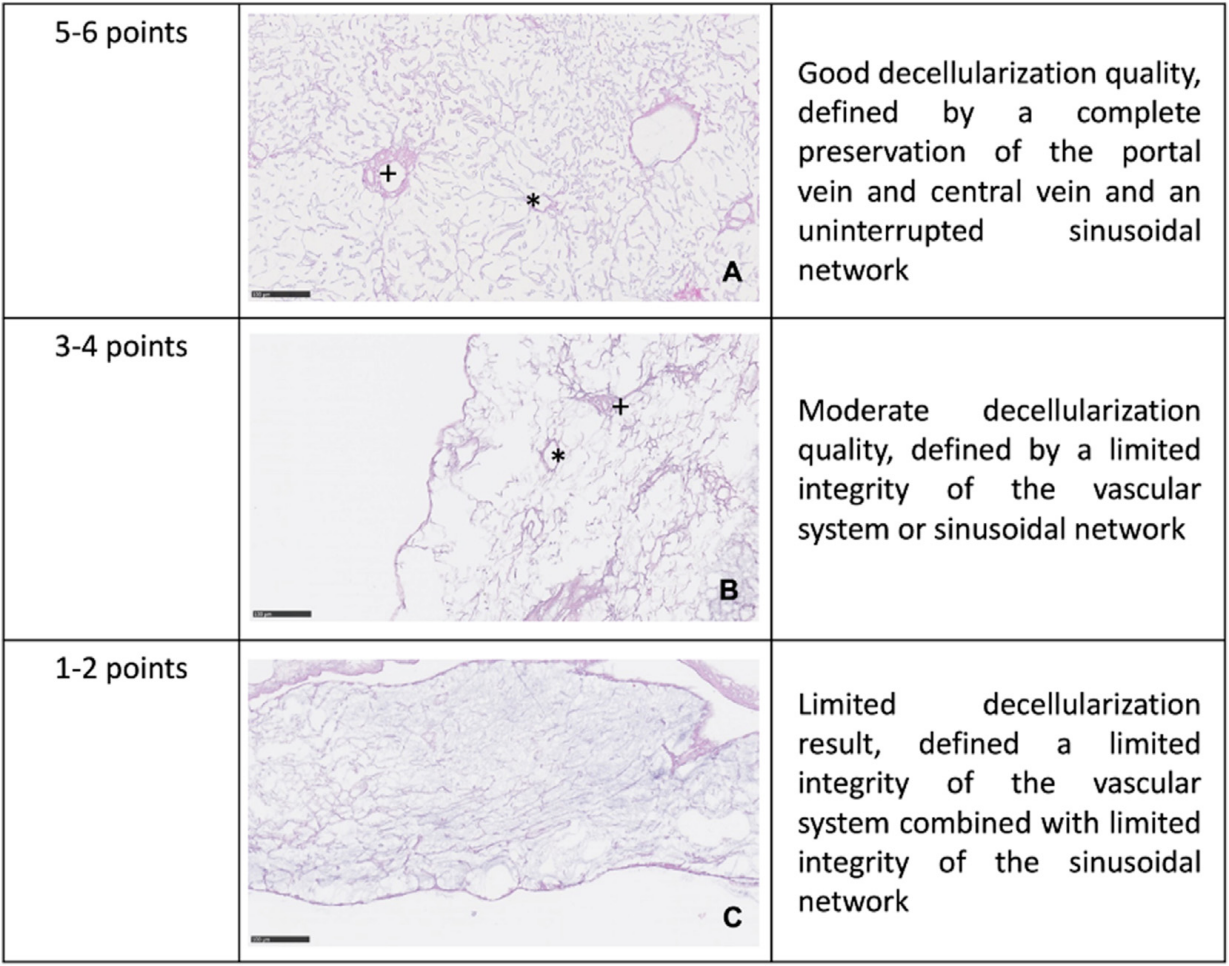
Grading of the histological decellularization score: The histological decellularization score was applied on all cell-free scaffold-sections: A (5–6 points): Good decellularization quality, defined by complete a complete preservation of the portal vein and central vein and an continuous sinusoidal network; B (3–4 points): Moderate decellularization quality, defined by a limited integrity of the vascular system or sinusoidal network; C (1–2 points): Limited decellularization result, defined by limited integrity of the vascular system combined with limited integrity of the sinusoidal network.

The histological decellularization score was applied on all cell-free scaffold-sections and based on six predefined criteria. The points of each criterion were added up to a score and used to grade the preservation level of microarchitecture (5–6 points: Good, shown in Figure 3A; 3–4 points: Moderate, shown in Figure 3B; 1–2 points: Limited, shown in Figure 3C).

1) Evaluation of portal vein integrity
  a) Presence of portal vein in HE staining (present: 1 point, absent: 0 points)
  b) Presence of continuous elastic fibers of the portal vein in EvG staining (present: 1 point, absent: 0 points)
2) Evaluation of central vein integrity
  a) Presence of central vein in HE staining (present: 1 point, absent: 0 points)
  b) Presence of continuous elastic fibers of the central vein in EvG staining (present: 1 point, absent: 0 points)
3) Evaluation of sinusoidal network integrity
  a) Presence of sinusoidal network (present: 1 point, absent: 0 points)
  b) If present, continuity of network structure (continuous: 1 point, interrupted 0 points)

#### 2.13.2 Immunohistochemical decellularization score

**Figure 4:**
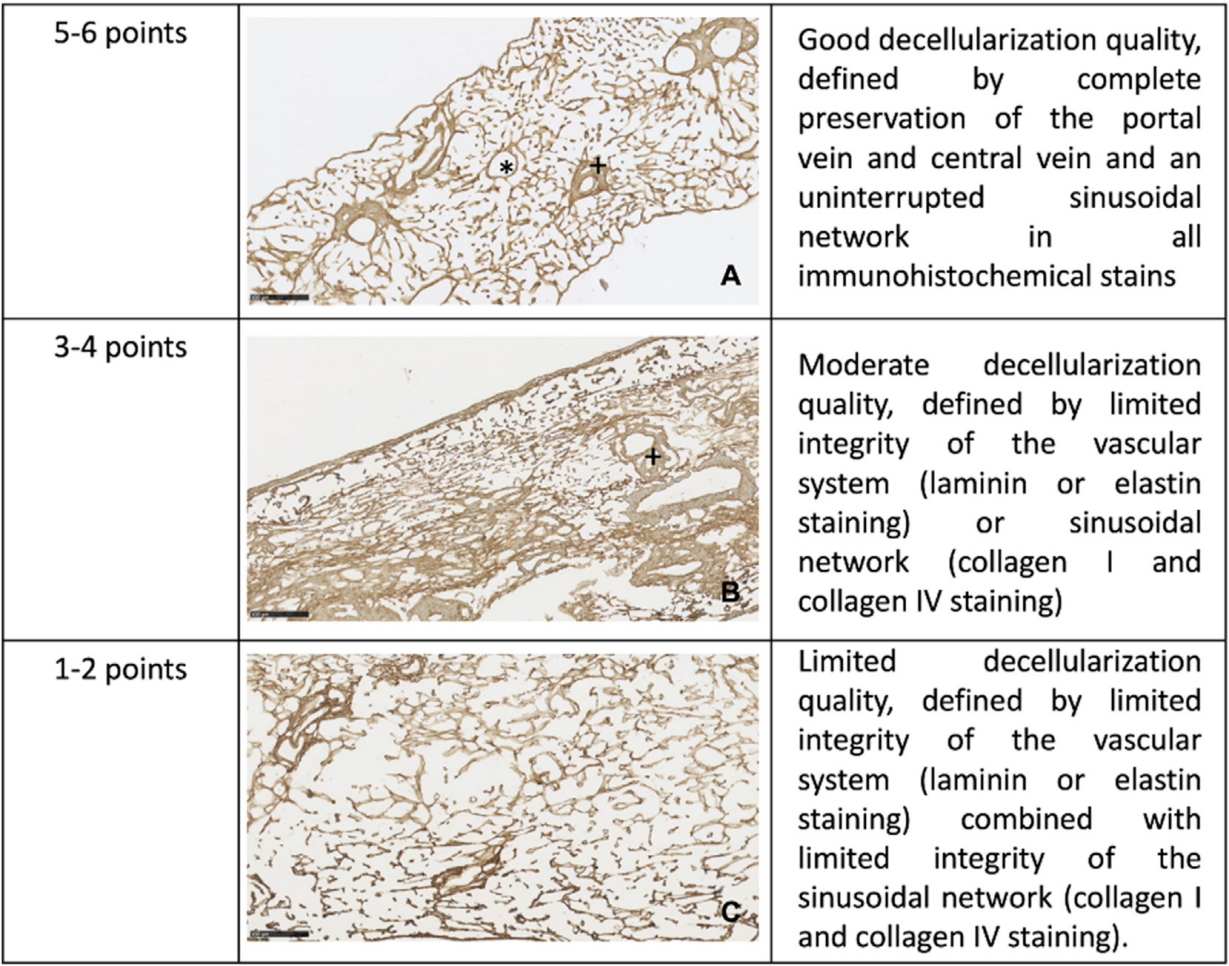
Grading of the immunohistochemical decellularization score: The immunohistochemicaldecellularization score applied on all cell-free scaffold-sections: A (5–6 points): Good decellularization quality, defined by complete preservation of the portal vein and central vein and an continuous sinusoidal network in all immunohistochemical stains; B (3–4 points): Moderate decellularization quality, defined by limited integrity of the vascular system (laminin or elastin staining) or sinusoidal network (collagen I and collagen IV staining); C (1–2 points): Limited decellularization quality, defined by limited integrity of the vascular system (laminin or elastin staining) together with limited integrity of the sinusoidal network (collagen I and collagen IV staining).

The immunohistochemical decellularization score was applied on one randomly selected cell-free scaffold-section of each organ and based on six predefined criteria. The points of each criterion were added up to a score and used to grade the preservation level of four selected matrix proteins (5–6 points: Good, shown in Figure 4A; 3–4 points: Moderate, shown in Figure 4B; 1–2 points: Limited, shown in Figure 4C).

1) Evaluation of portal vein integrity
  a) Presence of collagen I and collagen IV signals in the portal vein (present: 1 point, absent: 0 points)
  b) Presence of continuous elastic fibers and basal membrane of the portal vein in elastin as well as laminin staining (present: 1 point, absent: 0 points)
2) Evaluation of central vein integrity
  a) Presence of collagen I and collagen IV staining in the central vein (present: 1 point, absent: 0 points)
  b) Presence of continuous elastic fibers and basal membrane of the central vein in elastin as well as laminin staining (present: 1 point, absent: 0 points)
3) Evaluation of sinusoidal network integrity
  a) Presence of sinusoidal network in collagen I and collagen IV staining (present: 1 point, absent: 0 points)
  b) Continuity of the sinusoidal network in collagen I and collagen IV staining, if present (continuous: 1 point, interrupted: 0 points)

## 3 Ethics

All procedures, experiments, and housing of animals were carried out according to current German regulations and guidelines for animal welfare and to international principles of laboratory animal care. Furthermore, the study was approved by the State of Thuringia (Thueringer Landesamt fuer Verbraucherschutz, Thuringia, Germany (No. 02-074-14)).

## 4 Statistical analysis

The results of this experimental exploration study were evaluated qualitatively as described in the section macroscopical, microscopical and morphological assessment.

## 5 Results

### 5.1 Establishment of experimental groups

#### 5.1.1 Steatosis induction – fat diet (STEA)

The experimental induction of steatosis was achieved in 7 of 9 mice after 4 weeks of feeding with the methionine-choline-deficient and fat-enriched diet. The histological examination (HE-staining) of the steatosis tissue showed an increase in fat vacuoles (see arrow in Figure 8) compared to native liver tissue (shown in Figure 8). However, no signs of inflammation, such as an invasion of macrophages, were observed confirming the induction of non-alcoholic liver disease.

#### 5.1.2 Fibrosis induction – bile duct ligation (BDL)

Experimental induction of fibrosis was achieved in 9 of 9 mice within 4 weeks observation period. All animals recovered from the bile duct ligation procedure, indicated by a mild weight gain of approximately 17% compared to the starting body weight. The histological examination of the livers revealed an increase in the extracellular matrix around the portal field and massive bile duct proliferation, as expected after surgical induction of cholestasis (shown in Figure 8).

#### 5.1.3 Nodular regenerative hyperplasia – extended 90% partial hepatectomy (PH)

**Figure 5:**
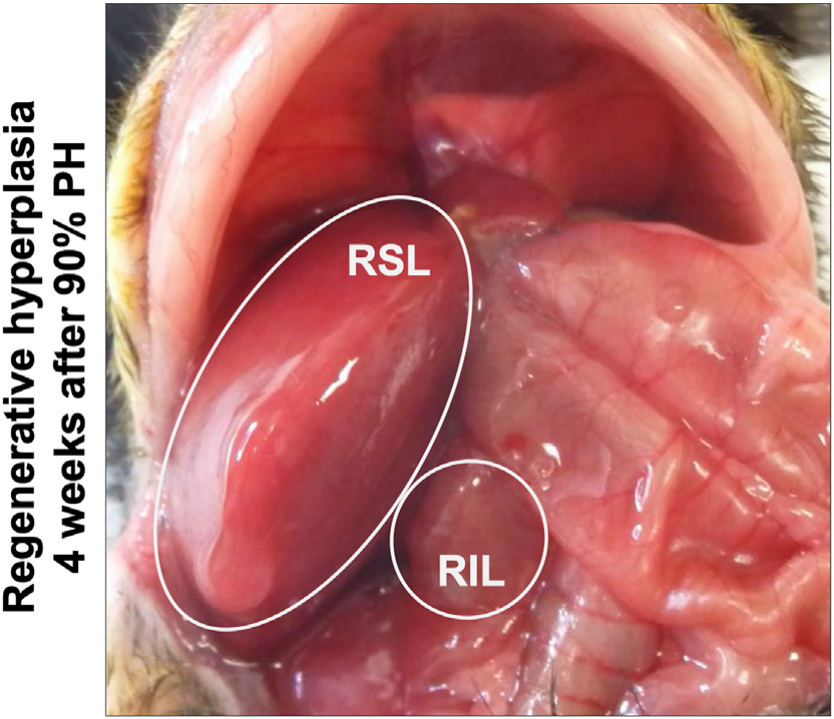
Intraoperative view after experimental induced nodular regenerative hyperplasia: The noular regenerative hyperplasia was induced and observed for at least 4 weeks after 90% partial hepatectomy. The image shows the remaining hypertrophied liver lobes, right superior lobe (RSL) and right inferior lobe (RIL) are marked with a white circle.

The nodular regenerative hyperplasia was achieved in 8 of 9 mice by performing a 90% partial hepatectomy. One animal died during the operative procedure as a result of an acute hemorrhage that could not be controlled. During liver explantation procedure, the increased liver volume of the regenerated right superior lobe (RSL) and the regenerated stump of the right inferior lobe (RIL) became visible (white circled shown in Figure 5). Due to the surgical resection of both large liver lobes (median and left lateral lobe), and the moderately sized right superior lobe, only one histological sample was taken from the regenerated right superior liver lobe. Lobular reorganization was indicated by an irregular nodular hyperplasia leading to an irregular lobular morphology with enlarged hepatocytes (shown in Figure 8).

### 5.2 Vascular system examination before and after decellularization

To visualize the vascular system of the liver before and after decellularization in each experimental group, the vascular trees of the contrasted livers and scaffolds were scanned by Tomoscope Duo CT.

Independent of the experimentally induced pathologies or decellularization procedure, the vascular tree of the liver was completely contrasted up to the lobular branches of the third order.

**Figure 6:**
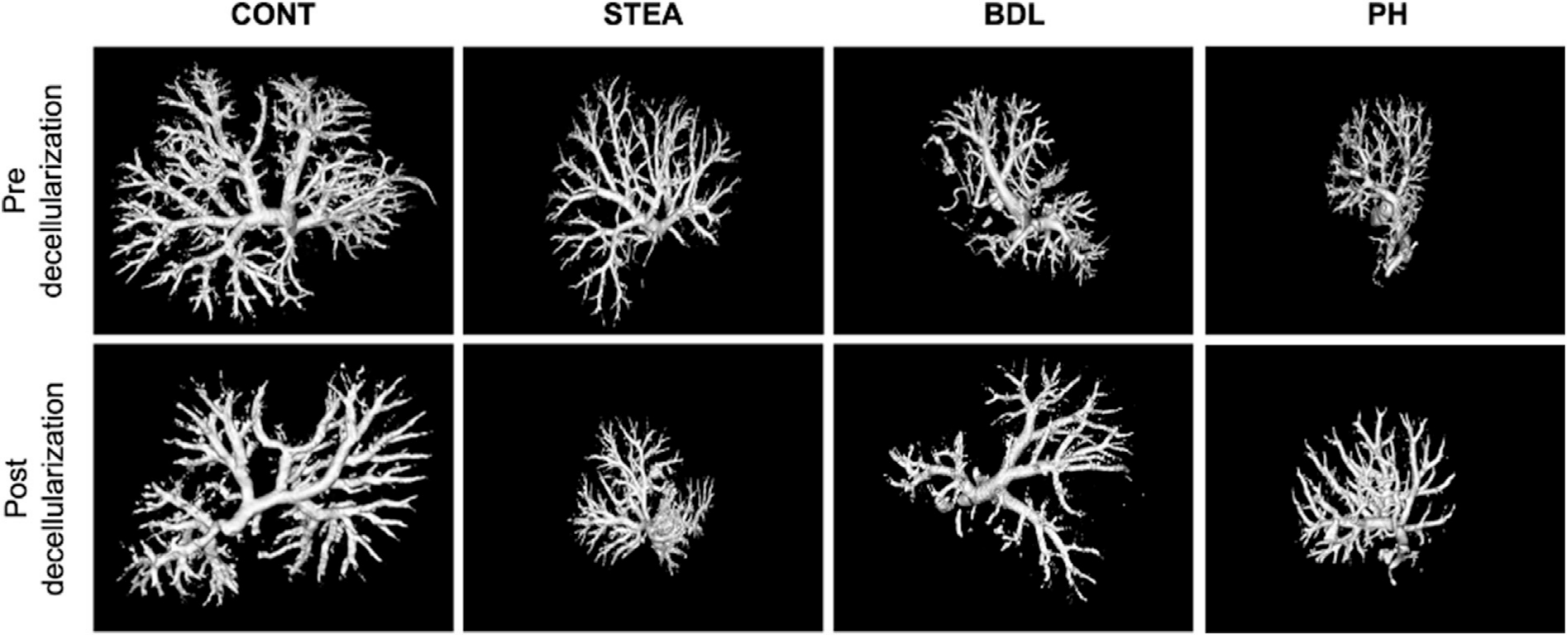
Vascular reconstruction of the portal vain: The integrity of the vascular tree before and after decellularization is demonstrated in all study groups (CONT: native, STEA: steatosis group, BDL: fibrosis group, PH: nodular regenerative hyperplasia group). For the analysis, the livers were injected with MICROFIL® and scanned by Tomoscope Duo CT (CT Imaging GmbH, Erlangen, Germany).

No extravasation of the radiopaque silicone rubber was observed in any of the experimental groups, leading to the conclusion that the vascular tree remained intact in all examined liver scaffolds (shown in Figure 6).

### 5.3 Macroscopic assessment

**Figure 7:**
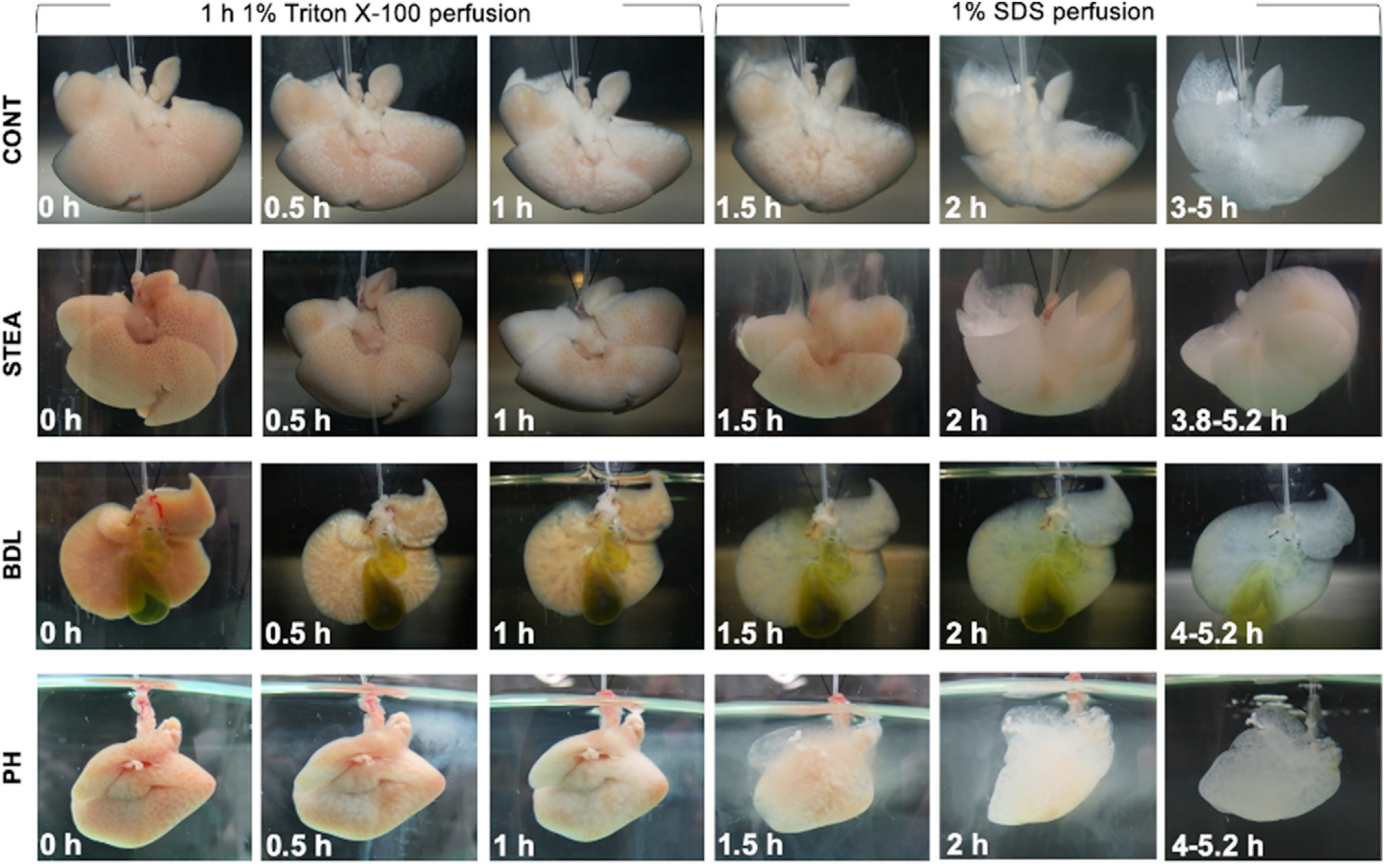
Representative course of the decellularization process: The course of decellularization process in all experimental groups (CONT: native, STEA: steatosis group; BDL: fibrosis group, PH: nodular regenerative hyperplasia group).

Independent of the underlying pathology, regions without tissue remnants and organs with good decellularization result according to the decellularization score were identified in all experimental groups (shown in Figure 7).

The frequency of organs with good decellularization result varied in the experimental groups (Table 1), (CONT: 3 out of 9 organs; STEA: 3 out of 7 organs, BDL: 3 out of 9 organs, PH: 2 out of 8 organs).

**Table 1:** Macroscopic decellularization quality: After the decellularization procedure, macroscopic quality of the scaffold was evaluated by the macroscopic decellularization score consisting of two criteria. The criteria were based on the distribution and extent of remnant hepatic parenchymal tissue and the distribution and extent of translucent areas.

Group	control group	STEA group	BDL group	90 % PH group
Score	4 1 1 1 3 4 4 3 3	3 3 4 4 4 3 3 - -	2 4 4 4 1 3 1 2 1	1 3 1 1 3 4 2 4 -
Median	3	3	2	2.5

The decellularized organs of the STEA and CONT group showed a median macroscopic decellularization score of 3 points. In contrast, the decellularized organs in the BDL and PH group achieved a median macroscopic decellularization score of 2 points and 2.5 points, respectively. In the PH group organs with macroscopically poor decellularized result were identified more often (3 out of 8 organs with a macroscopic decellularization score of 1 point).

The highest number of organs with macroscopically good decellularized result was found in the STEA group (3 organs with a macroscopic decellularization score of 4 points; 4 organs with a macroscopic decellularization score of 3 points), possibly due to the increased stiffness of the tissue facilitating a more homogenous perfusion.

The course of decellularization differed slightly between the groups. As expected, perfusion with Triton X-100 led only to a limited change in the macroscopic appearance of the organs in all groups. In contrast, after approximately 30 min of perfusion with SDS (shown in Figure 6), the organs started to get more and more translucent. Interestingly, the time to achieve a similar level of decellularization varied according to the pathological alteration when comparing the different groups. Organs from the control group were decellularized most efficiently in approximately 3.8 h ± 0.73. In contrast, the decellularization process in organs of all other experimental groups was less effective and took longer (STEA (4.9 h ± 0.5), BDL (4.8 h ± 0.6), and PH (5.1 h ± 0.4)) (Table 2).

**Table 2:** Duration of the decellularization process: The duration of the decellularization process was determined in all groups as the time until the liver gets translucent or no macroscopic changes were seen for a period of more than 30 min.

Group	control group	STEA group	BDL group	90 % PH group
Time (h)	5 3.2 3.5 4.2 4 4.2 3 3 4.3	5 5 5.2 4.8 3.8 5 5.2 - -	5.2 4 4 4 5.2 5.2 5.2 5.2 5.2	5.2 5.2 5.2 5.2 5.2 5.2 5.2 4 -
Average (±STD)	3.8 (±0.73)	4.9 (±0.5)	4.8 (±0.6)	5.1 (±0.4)

### 5.4 Microscopic assessment

According to the sequential morphological workflow, 145 decellularized scaffold-sections without macroscopically visible tissue remnants were taken from organs and subjected to HE- and PAS-staining.

Cell nuclei or cytoplasmic glycogen were microscopically visible in 57 scaffold-sections, including all sections from the 3 organs of the PH group with poor macroscopic decellularization result.

According to the *sequential morphological workflow*, all sections with residual cells, were excluded from further analysis. The remaining 88 decellularized scaffold-sections (CONT: 15 sections; STEA: 38 sections, BDL: 30 sections, PH: 5 sections) were subjected to the morphological assessment.

### 5.5 Morphological assessment

The morphological assessment identified the level of preserved liver-specific microarchitecture and matrix proteins in cell-free scaffold-sections after decellularization procedure. For this purpose, the cell-free sections were subjected to histological as well as immunohistochemical staining and evaluated using the morphological decellularization scores.

**Figure 8:**
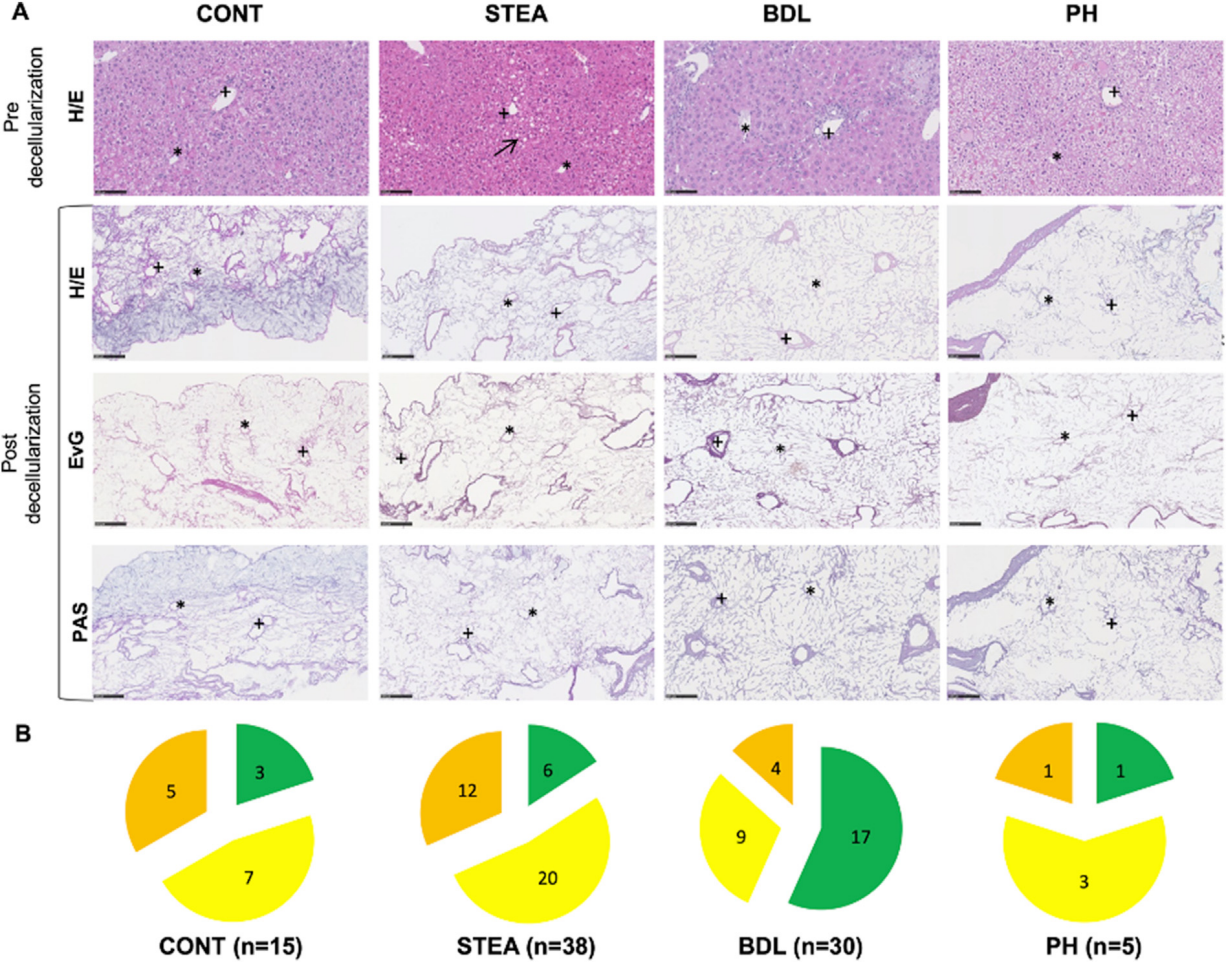
Examples of the histological structure in all experimental groups and the relative distribution according to the histological decellularization score: A: Histological work-up of all experimental groups (CONT: native, STEA: steatosis group; BDL: fibrosis group, PH: nodular regenerative hyperplasia group) before and after good decellularization (+: portal field; ∗: central vein; arrow: fat vacuoles). B: Distribution of the decellularization quality based on the quantitative microscopic decellularization score (green = good decellularization quality, yellow = moderate decellularization quality, orange = limited decellularization quality) for all experimental groups.

Using the histological decellularization score, preservation of scaffold microarchitecture was demonstrated in sections of all experimental groups, albeit at different frequencies. Scaffold-sections with good histological decellularization result were mostly found in the BDL group (17 out of 30 sections) and in the STEA group (6 out of 38 sections). In contrast, good histological decellularization result was only observed in only 3, respectively, 1 scaffold-sections of the CONT group and PH group. The remaining scaffold-sections showed a moderate or limited decellularization result (CONT: 7 out of 15 sections with moderate decellularization result, 5out of 15 sections with limited decellularization result; STEA: 20 out of 38 sections with moderate decellularization result, 12 out of 38 sections with limited decellularization result; BDL: 9 out of 30 sections with moderate decellularization result, 4 out of 30 sections with limited decellularization result; PH: 3 out of 5 sections with moderate decellularization result, 1 out of 5 section with limited decellularization result) (shown in Figure 8 B).

Using the immunohistochemical decellularization score, preservation of the selected matrix proteins was also demonstrated in all experimental groups, but again at different frequencies.

**Figure 9:**
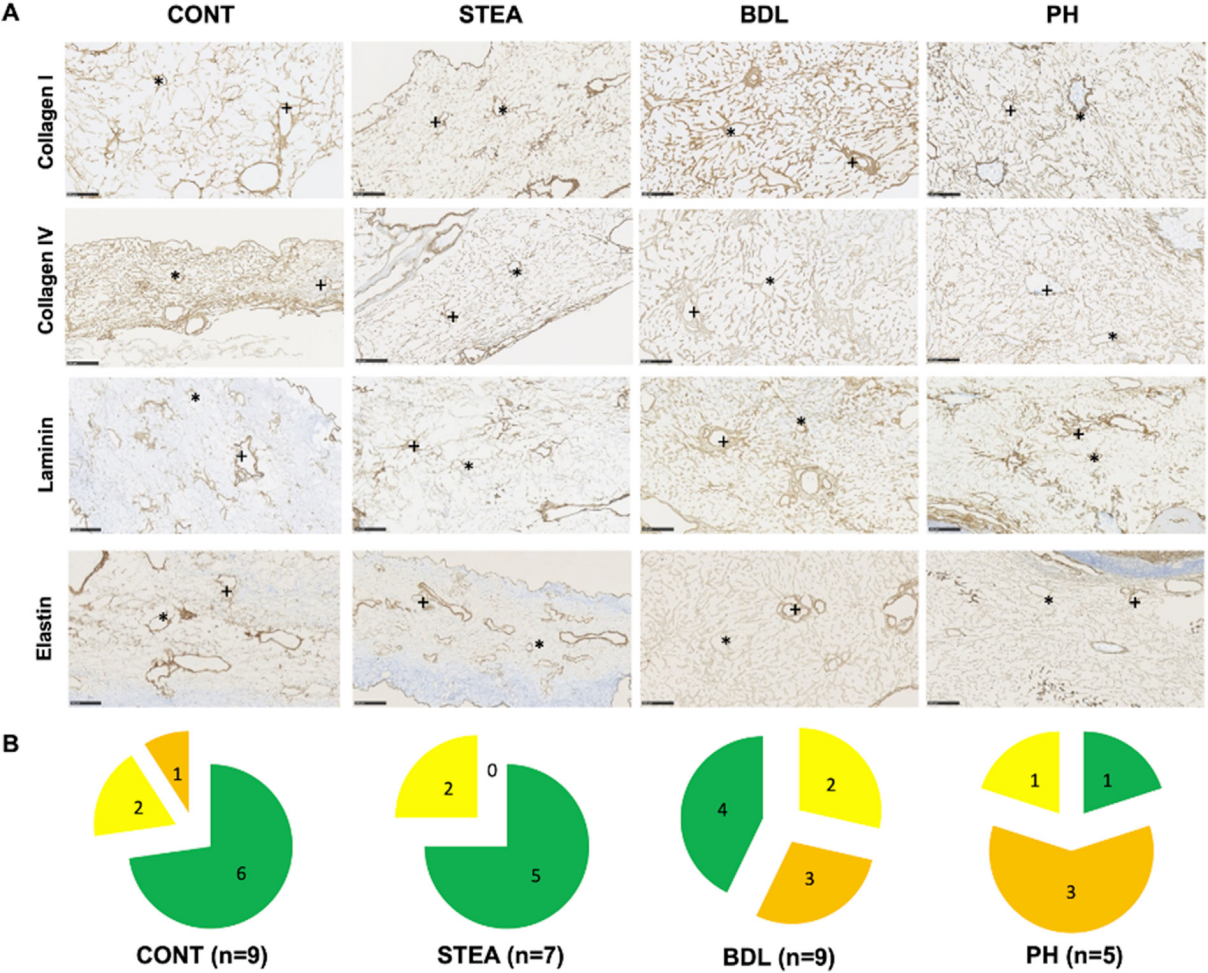
Examples of the histological structure in all experimental groups and the relative distribution according to the immunohistochemical decellularization score: A: Immunohistochemical work-up of all experimental groups (CONT: native, STEA: steatosis group; BDL: fibrosis group, PH: nodular regenerative hyperplasia group) following good decellularization (+: portal field; ∗: central vein). B: Distribution of the decellularization result based on the quantitative immunohistochemical decellularization score (green = good decellularization quality, yellow = moderate decellularization quality, orange = limited decellularization quality) for all experimental groups.

A good immunohistochemical decellularization result, indicated by the immunohistochemical detection of preserved matrix proteins in the microarchitecture, was obtained in more than 50% of the scaffold-sections (CONT: 6 out of 9 sections; STEA: 5 out of 7 sections; BDL: 4 out of 9 sections; PH: 1 out of 5 sections). The highest rate of scaffold-sections with only limited immunohistochemical decellularization result was identified in the PH group (3 out of 5 sections) (shown in Figure 9).

## 6 Discussion

We presented our sequential morphological workflow for the evaluation of cell-free murine scaffold sections. This morphological workflow takes three different aspects into account: a macroscopic, microscopic and morphological evaluation. The application of this workflow allowed a structured morphological analysis of the cell-free liver scaffold and identified the tissue samples with best preserved microarchitecture and matrix composition, as assumingly needed for further reseeding and repopulation experiments.

Based on the assumption that only marginal human donor organs with morphological changes will be available for allogeneic tissue engineering [22], we induced similar structural alterations [23, 24, 25] in mice. We used an established perfusion protocol for the subsequent decellularization procedure [3, 18] and investigated the effect of the induced structural alterations on the decellularization process and quality. This experimental approach allowed us to demonstrate the feasibility of decellularization in both, native livers and structurally altered organs. Furthermore, using our sequential morphological workflow, we were able to compare morphological decellularization result in four different experimental groups. The frequency of organs with good decellularization results varied in the experimental groups. The STEA group and the BDL group obtained the highest number of scaffold samples with good macroscopic, microscopic and morphological decellularization results. In contrast, scaffold-sections of the PH group were mostly of limited morphological decellularization quality.

We speculate that the differences in the structural quality of the decellularized scaffold could be caused by the inhomogeneous decellularization process. The inhomogeneous decellularization, in turn, could be due to the experimentally induced pathology of the organs and the resulting heterogeneous organ perfusion.

Our sequential morphological workflow improves the classification of the microarchitecture preservation level in decellularized murine liver scaffolds.

We consider this to be helpful for the selection of cell-free and well-preserved scaffold-sections to be used for the subsequent investigation of cell scaffold interaction.

Nevertheless, any morphological scoring system is limited by the interobserver variability [26, 27]. Digital image analysis of decellularized liver scaffolds is needed to reduce this interobserver variability in the future. For certain well selected quantification needs, image analysis algorithms are already available. One example is the automatic quantification of the hepatic fat content based on the automatized detection of the relative surface occupied by the fat vacuoles in the hepatocytes compared to the total surface of the section. For this purpose either images from fresh frozen sections stained by oil red O [28] or from hematoxylin-and-eosin-stained paraffin sections [29] are used. Based on a threshold analysis, color differences between the fat vacuoles and the surrounding tissues are detected. Classification rules are used to identify the fat vacuoles and determine their size and number.

However, automated image analysis of liver scaffolds represents a much greater challenge. Due to the complex nature of the underlying structure of e.g. the network and the impact of the decellularization on this delicate network, quantification cannot be achieved with simple threshold analysis.

However, the prerequisite for the development of image analysis algorithm is the identification of potentially relevant structures and parameters to be quantified.

We suggest to use parameters such as the integrity of the sinusoidal network as basis for quantification. However, the sinusoidal network on the liver is not one network but consists of multiple small networks in each liver lobule. Therefore, network analysis calls for automatic identification of hepatic lobuli followed by a quantification of network features, e.g. length of sinusoidal structures. A first algorithm was presented recently by our group using porcine scaffolds [12]. Lobule identification in pig scaffolds is facilitated by the septa surrounding each lobule. However, murine, rat and human livers do not have any septa around the lobules, which represents an additional and so far unsolved challenge for software developers. Our results stress the importance of using these networks structures for a quantitative computer-based analysis.

A further alternative to morphological quality determination is the examination of the decellularization result using non-invasive parameters. Recently, Geerts *et al.* suggested subjecting the scaffold to CT-scanning [16] for the determination of the Hounsfield units as an indirect measure of the extent of decellularization. However, this approach is of limited value since it does not allow an optimal assessment of the organ-specific microarchitecture.

In contrast, Uzarski *et al.* proposed to assess the decellularization process as an indirect indicator of the resulting scaffold quality. Therefore, they measured the perfusion pressure resistance over time during decellularization and observed a reduced perfusion pressure at the end of the decellularization process [30]. This idea was further extended by Nishii *et al.*, who investigated the hemodynamic changes in the vascular system in native and decellularized livers based on a computer-assisted liver perfusion model [31]. This model was created to predict the decreased vascular resistance (up to 81%) as well as the decreased vascular pressure (more than 5 times) caused by the decellularization process. Both studies support the inclusion of non-invasive pressure monitoring in the quality control evaluation of the decellularization process.

However, in our study, decellularization did not always proceed homogenously in the organ, making a pressure-dependent assessment also difficult. Parenchymal remnants were detected in livers from all experimental groups, albeit at different frequencies. Residual parenchymal islands were more often detected in the CONT and PH groups than in the STEA and BDL groups. The key difference between these groups is the stiffness of the organ, corresponding to the histologically confirmed increased fibrosis and the fat content of the liver. Due to the fragility of the murine liver, especially the native liver, even small changes in positioning of the organ during decellularization can cause a kinking of the intrahepatic vessels and impair perfusion. We speculate that differences in the loss of stiffness during perfusion might further promote perfusion irregularities and subsequently influence the homogeneity of the decellularization throughout the organ. In this case, decellularization of structurally altered livers would possibly require an adaptation of the decellularization protocol as well as the criteria for termination of the perfusion process.

## 7 Conclusion

Our sequential morphological workflow allowed the analysis of decellularized liver sections, irrespectively of eventually underlying pathological alterations. Furthermore, using our proposed sequential morphological workflow, we were able to detect tissue sections with well-preserved hepatic microarchitecture, as needed for further repopulation experiments.

## Declarations

### Author contribution statement

Philipp Felgendreff, Claudia Schindler, Uta Dahmen: Conceived and designed the experiments; Analyzed and interpreted the data; Wrote the paper.

Franziska Mussbach: Conceived and designed the experiments; Performed the experiments; Analyzed and interpreted the data.

Chichi Xie: Performed the experiments; Analyzed and interpreted the data; Wrote the paper.

Felix Gremse: Analyzed and interpreted the data.

Utz Settmacher: Analyzed and interpreted the data; Wrote the paper.

### Funding statement

This work was supported by the Else-Kröner-Fresenius Foundation” (KFK_2015_Kolleg.15-02).

### Data availability statement

Data will be made available on request.

### Declaration of interests statement

The authors declare no conflict of interest.

### Additional information

No additional information is available for this paper.
